# The correlation between vitamin D and the occurrence of peripheral neuropathy induced by paclitaxel chemotherapy

**DOI:** 10.3389/fmed.2024.1466049

**Published:** 2024-09-24

**Authors:** Jialei Zhang, Xiaoling Zhang, Jie Wu

**Affiliations:** ^1^Department of Pain Treatment, Changzhi People’s Hospital Affiliated to Changzhi Medical College, Changzhi, China; ^2^Laboratory Animal Center, Shanxi Medical University, Taiyuan, China; ^3^Department of Oncology, Changzhi People’s Hospital Affiliated to Changzhi Medical College, Changzhi, China

**Keywords:** vitamin D, neuropathic pain, paclitaxel-induced peripheral neuropathy, pain, chemotherapy

## Abstract

**Introduction:**

Paclitaxel, a widely used chemotherapeutic agent for various cancers, induces peripheral neuropathy (PIPN) in approximately 80% of patients, severely affecting their quality of life. The role of vitamin D in pain perception has gained attention, but its correlation with PIPN remains unclear.

**Methods:**

This study included 129 cancer patients who received adjuvant paclitaxel chemotherapy from January to June 2023. Neuropathic pain was assessed using the Douleur Neuropathique 4 Questions (DN4) questionnaire, and serum levels of vitamin D and glutathione (GSH) were measured to explore the correlation between vitamin D levels and neuropathic pain induced by paclitaxel chemotherapy.

**Results:**

The results showed a negative correlation between vitamin D deficiency and the occurrence of neuropathic pain (Spearman correlation coefficient of −0.324, *P* < 0.001). The receiver operating characteristic (ROC) curve analysis revealed that the area under the vitamin D curve for neuropathic pain was 0.681. Furthermore, after paclitaxel chemotherapy, there was a significant decrease in GSH levels in the serum of patients, with a more pronounced decline in the vitamin D-deficient group.

**Discussion:**

The findings of this study indicate that higher levels of vitamin D are negatively associated with the occurrence of paclitaxel-induced neuropathic pain, suggesting that vitamin D might protect against oxidative stress. This discovery is significant for clinical treatment as it may help physicians better understand the mechanisms of pain during paclitaxel therapy and provide new strategies for the prevention and treatment of such pain. It also suggests that modulating vitamin D levels could reduce the neurotoxicity of paclitaxel, thereby improving patients’ quality of life and treatment compliance.

## Introduction

As a first-line clinical anti-tumor drug, paclitaxel has excellent therapeutic effects on cancers such as breast cancer, ovarian cancer, fallopian tube cancer, lung cancer, and gastric cancer. It can bind to *β*-tubulin to stabilize the microtubule structure, inhibit cell mitosis, thereby causing mitochondrial damage and cell apoptosis (1). Chemotherapy-induced peripheral neuropathy(CIPN) is one of the most common adverse reactions that limit the use of anti-tumor drugs. Paclitaxel-induced peripheral neuropathy(PIPN) usually occurs 24–72 h after administration, with an incidence rate as high as 87% (2). Clinically, it mainly manifests as numbness, weakness, and burning sensation of the limbs, accompanied by tingling or even autonomic nerve dysfunction (3). Compared with other peripheral neuropathies (such as painful diabetic polyneuropathy), patients with PIPN may experience more severe pain, affecting both hands and feet, along with heat or cold hyperalgesia (4), and the progression of symptoms is also faster.

Epidemiological data indicate that vitamin D deficiency is more prevalent among the elderly population in our country compared to deficiencies in other trace elements (5, 6). Our research team’s study shows that vitamin D deficiency in elderly patients during the perioperative period can lead to increased expression of central nervous system inflammatory factors, thereby causing postoperative cognitive dysfunction (7). Adequate vitamin D is not only important for bone health but also has a good therapeutic effect on various chronic pains. Studies have shown that vitamin D can have a significant therapeutic effect on the treatment of diabetic neuropathy (8), postherpetic neuralgia (9), and other neuropathic pains by promoting the expression of nerve growth factor (NGF) in nerve cells (10), inhibiting inflammation and immune regulation (11), and antiviral effects (12). However, apart from diabetic neuropathy, there is less research on the correlation between vitamin D and other types of neuropathic pain (NP), and there is a lack of high-quality randomized controlled trials. At the same time, the specific mechanism of vitamin D in treating NP is still unclear, and the evaluation of efficacy and mechanism research requires further advancement through more clinical trials and basic research.

This study intends to assess the occurrence and degree of neuropathic pain in postoperative patients who receive adjuvant chemotherapy with paclitaxel, utilizing the Douleur Neuropathique 4 Questions (DN4) questionnaire. Concurrently, the research will analyze the levels of vitamin D and glutathione in serum to explore the correlation between vitamin D levels and the neuropathic pain induced by paclitaxel chemotherapy, as well as the potential underlying mechanisms. The goal is to predict effectively and intervene early to mitigate the adverse effects that paclitaxel chemotherapy may have on patients.

## Experimental procedures

### Study design

This study is a single-center prospective cohort. By comparing the serum vitamin D levels of patients undergoing paclitaxel chemotherapy with the incidence of neuropathic pain, we investigate the potential correlation between these two factors. This experiment was approved by the Medical Ethics Committee of Changzhi City People’s Hospital (Approval Number: 2023 K06), all experiments were performed in accordance with relevant guidelines and regulations, and is registered with the Chinese Clinical Trial Registration Center (Registration Number: ChiCTR2200065351). All participating patients have signed informed consent forms.

### Patients

Patients who received postoperative adjuvant chemotherapy with paclitaxel for breast, lung, and cervical cancer at the Oncology Department of Changzhi City People’s Hospital between January 2023 and June 2023 were selected. The inclusion criteria were age between 65 and 75 years old, no abnormalities in liver and kidney functions, and the ability to cooperate in completing all test questionnaires. The exclusion criteria were: severe circulatory and respiratory system diseases; neurological or psychiatric disorders that make it impossible to cooperate with the trial; visual and auditory impairments that prevent completion of the tests; a history of severe drug allergies; and participation in other clinical trials.

### Chemotherapy regimen

The chemotherapy regimen for cervical cancer that we used consists of paclitaxel at a dosage of 175 mg/m^3^, administered intravenously over 3 h, repeated every 3 weeks for a total of six cycles. Additionally, to prevent the emergence of pain tolerance and other such conditions caused by multiple chemotherapy sessions, this experiment only tests and compares the results after the first chemotherapy.

### Neuropathic pain assessment

Screening for post-chemotherapy pain using the DN4 neuropathic pain scale to differentiate neuropathic pain from non-neuropathic pain. The DN4 scale consists of seven self-assessment symptom items and three clinical examination items, each scored at one point, with a maximum total score of 10 points. A total score of four or above can diagnose neuropathic pain. Research indicates (13), the DN4 scale has a sensitivity of 83% and a specificity of 90% for diagnosing neuropathic pain, and it is advantageous for its simple operation and ease of understanding. Patients are categorized into the Neuropathic Pain Group (PIPN Group) and the Non-Neuropathic Pain Group (Non-PIPN Group) based on the presence of neuropathic pain.

### Observational indicators

Document the general information of patients, including age, BMI, cancer pathology staging, hypertension, diabetes, coronary heart disease, and chronic obstructive pulmonary disease (COPD). On the morning before chemotherapy, blood is collected in a fasting state to measure vitamin D and glutathione (GSH) levels in the blood using a blood cell analyzer. VAS pain scores are assessed 2 h after the completion of chemotherapy, and blood samples are taken again to test for GSH levels. A comparison of GSH levels between pre- and post-chemotherapy, as well as between the Neuropathic Pain Group (NP group) and the Non-Neuropathic Pain Group (Non-NP group), is carried out.

According to the “Guidelines for the Treatment and Prevention of Vitamin D Deficiency” published by the American Endocrine Society (14), and the “Expert Consensus on the Clinical Application of Vitamin D in the Elderly (2018)” published by the Osteometabolic Disease Study Group of the Geriatric Medicine Branch of the Chinese Medical Association (15), the levels of Vitamin D (VD) are defined as follows: Adequate: >20 ng/mL, Insufficient: 12–20 ng/mL, Deficient: <12 ng/mL. This study divided the participants into the Deficient group and the Non-deficient group based on the levels of VD.

### Statistical analysis

Based on Vitamin D (VD) levels before chemotherapy, patients were divided into the Deficient group and the Non-deficient group. Patients were also categorized into the Neuropathic Pain group (PIPN group) and the Non-Neuropathic Pain group (Non-PIPN group) depending on whether they developed neuropathic pain. Data analysis was conducted using the SPSS Statistics 23.0 software. Measurement data are presented as mean ± standard deviation (*x̄*±*s*), count data as median and interquartile range, and categorical data as number and percentage. Continuous variables with normal distribution were analyzed using the independent samples *t*-test, continuous variables and ordinal data not normally distributed were analyzed using the Mann–Whitney U test, and categorical data were analyzed using the chi-square test, Fisher’s exact test, or continuity correction chi-square test as appropriate. Binary Logistic regression analysis was performed to identify potential factors associated with the occurrence of neuropathic pain. The Receiver Operating Characteristic (ROC) curve was applied to analyze the predictive value of Vitamin D levels for the development of paclitaxel-induced neuropathic pain. A *p* value of less than 0.05 was considered statistically significant.

## Results

### Comparison of general conditions

From January 2023 to June 2023, a total of 382 postoperative breast cancer, lung cancer, and cervical cancer patients in our hospital’s oncology department underwent adjuvant chemotherapy with paclitaxel. To eliminate all confounding factors, we selected only cervical cancer patients as the subjects of our study. Ultimately, 129 patients were enrolled, of which 85 were patients with vitamin D deficiency (VD < 12 ng/mL) and 44 were non-deficient patients (VD > 12 ng/mL). There were no significant differences between the vitamin D deficient group and the non-deficient group in terms of age, gender, BMI, and pre-chemotherapy GSH levels. However, there were statistically significant differences between the two groups in the occurrence of neuropathic pain (NP) after chemotherapy, GSH levels, and VAS scores. Patients were divided into the NP group and the Non-NP group based on the occurrence of NP. There were no significant differences between the two groups in age, gender, BMI, and pre-chemotherapy GSH levels (*p* > 0.05), but there were significant differences in VD levels, post-chemotherapy GSH test results, and VAS scores (*p* < 0.05) (Table 1).

**Table 1:** Demographic and baseline characteristics.

	All patients *n* = 129	VD deficiency *n* = 85	No VD deficiency *n* = 44	*p*-value
Age, year	71.57 ± 3.87	71.03 ± 3.66	72.10 ± 4.07	0.290
male, *n* (%)	41 (31.8)	26 (30.6)	15 (34.1)	0.685
BMI, kg/m^2^	23.37 ± 2.44	22.93 ± 2.60	23.81 ± 2.23	0.164
Chronic smoking, *n* (%)	21 (16.3)	13 (15.3)	8 (18.2)	0.674
Alcoholism, *n* (%)	10 (7.7)	8 (9.4)	2 (4.5)	0.327
**Comorbidity, *n* (%)**				
Hypertension	64 (49.6)	40 (47.0)	24(54.5)	0.420
Diabetes mellitus	38 (29.4)	26 (30.6)	12(27.3)	0.695
Coronary heart disease	15 (11.6)	9 (10.6)	6(13.6)	0.609
Chronic lung diseases	8 (6.2)	6 (7.0)	2(4.5)	0.575
History of surgery, *n* (%)	16 (12.4)	10 (11.8)	6(11.4)	0.760
Vitamin D concentration, ng/mL	11.65 ± 3.47	9.66 ± 1.25	15.61 ± 3.05	<0.001
Serum GSH concentration, μg/mL	336.73 ± 17.39	341.19 ± 18.87	334.51 ± 16.39	0.163

### DN4 scores

Statistical analysis was performed on the DN4 scores of all patients, and they were divided into PIPN and Non-PIPN groups based on the results, showing a significant statistical difference between the two groups (*p* < 0.001). Additionally, a comparison of DN4 scores between patients with vitamin D deficiency and those without deficiency was conducted, and a significant statistical difference was also observed between these two groups (*p* = 0.273) (Table 2).

**Table 2:** DN4 scores statistics.

	DN4 scores	*p*-value
VD deficiency (*n* = 85)	5.09 ± 2.37	0.002
No VD deficiency (*n* = 44)	3.73 ± 2.37	
PIPN (*n* = 77)	6.38 ± 1.59	<0.001
Non-PIPN (*n* = 52)	2.07 ± 0.79	

### Neuropathic pain patient statistics and changes in GSH levels

**Figure 1:**
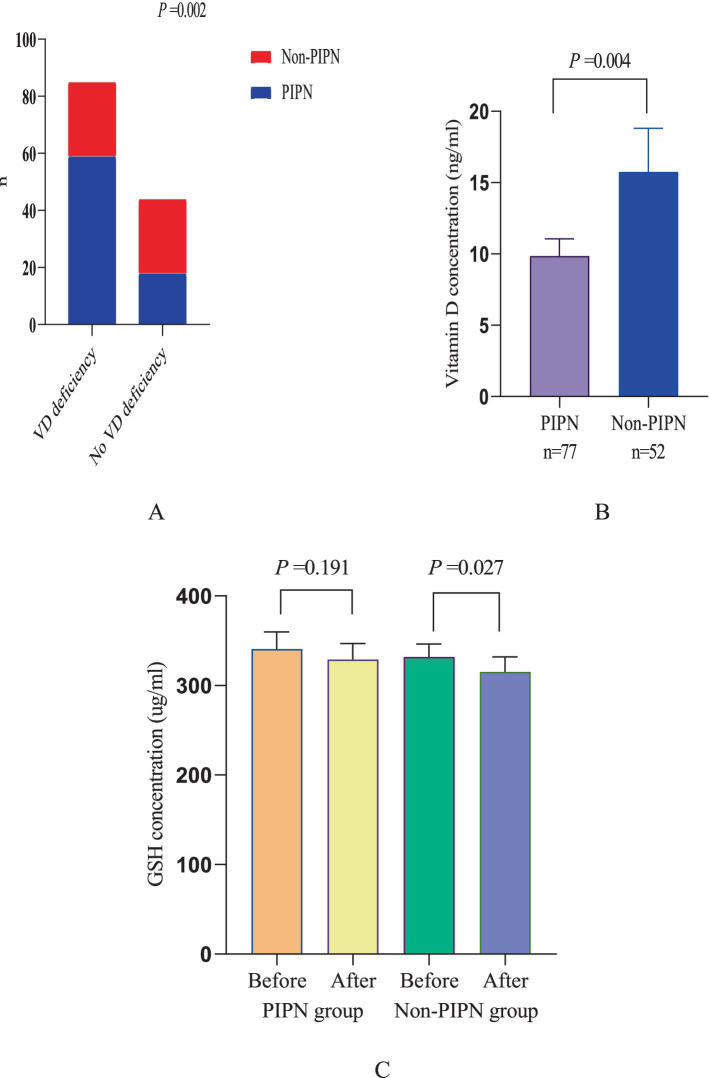
Neuropathic pain patient statistics and changes in GSH levels. **(A)** Comparison between PINP patients and non-PINP patients among patients with and without vitamin D deficiency. **(B)** Comparison of vitamin D levels between PINP patients and Non-PINP patients. **(C)** Comparison of GSH levels before and after paclitaxel chemotherapy between PINP patients and Non-PINP patients.

Among all patients, a total of 77 (59.7%) developed neuropathic pain, of which 59 cases occurred in patients with preoperative vitamin D deficiency (69.4%), and 18 cases occurred in patients without vitamin D deficiency (40.9%). Additionally, this study analyzed the trend of changes in GSH levels in the serum of patients before and after chemotherapy. It was found that there was no significant difference between patients with and without preoperative vitamin D deficiency (*p* = 0.273). After paclitaxel chemotherapy, GSH levels significantly decreased, and the decrease was more pronounced in patients with vitamin D deficiency compared to those without deficiency (*p* = 0.017) (Figure 1).

### Analysis of the correlation between vitamin D levels and the occurrence of neuropathic pain

Using rank correlation analysis to examine the correlation between vitamin D levels and the occurrence of paclitaxel-induced neuropathic pain, the results showed that with a two-tailed confidence level of 0.01 for the two variables, the Spearman correlation coefficient was −0.324, and *p* < 0.001, indicating that there is a certain correlation between the two variables, and it is a negative correlation. This means that the higher the level of vitamin D, the lower the probability of paclitaxel-induced neuropathic pain occurring.

### Confounding factor analysis

Using binary Logistic regression analysis, univariate analysis was conducted on count data (gender, smoking, alcohol consumption, and coexisting diseases such as hypertension, diabetes, coronary heart disease, chronic obstructive pulmonary disease), and significance tests were performed on measurement data (VD, age, BMI, and GSH). The results showed that the VD level before chemotherapy is related to the occurrence of neuropathic pain (OR: 0.826, 95% CI: 0.740–0.922, *p* = 0.001).

### Receiver operating characteristic curve

**Figure 2:**
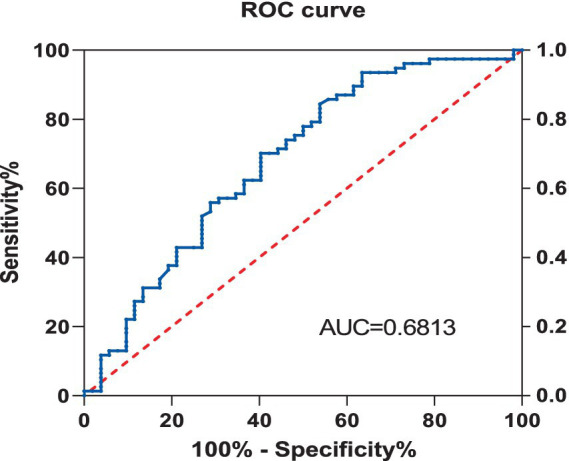
ROC curve for the predictive value of VD in NP.

Using the Receiver Operating Characteristic (ROC) curve, the predictive value of Vitamin D for Neuropathic Pain (NP) was explored. The analysis showed that the area under the Vitamin D curve for NP was 0.681, with a sensitivity of 84.4% and a specificity of 46.2%, and the 95% Confidence Interval (CI) was 0.585–0.778, with a *p* value of less than 0.001 (Figure 2).

## Discussion

Neuropathic pain (NP) is pain that arises as a direct consequence of a lesion or disease affecting the somatosensory system (16). NP can be caused by a variety of injuries to the peripheral or central nervous system, including metabolic disorders, infections, trauma, inflammation, and neurotoxic substances. In the treatment guidelines for NP, anticonvulsants, and tricyclic antidepressants are recommended as first-line medications, with opioids and lidocaine patches as second-line options (17). However, the therapeutic effects of these drugs are often suboptimal and are associated with varying degrees of adverse reactions, and there is still a lack of effective treatment for some refractory NP. The treatment of NP has become a global challenge mainly due to its diverse etiologies and complex mechanisms. The pathogenesis of NP has not been fully elucidated to date.

Paclitaxel-induced peripheral neuropathy may be caused by the destruction of axonal microtubule structures and the toxic effects of mitochondria in primary afferent neurons (18). Studies have shown that paclitaxel can alter the integrity and function of mitochondria, causing transient releases of calcium ions in the mitochondria, leading to the depolarization of the mitochondrial membrane and the opening of the mitochondrial permeability transition pore (mPTP), resulting in mitochondrial swelling in C-fibers and myelinated axons (19). In addition, paclitaxel can reduce the activity of mitochondrial respiratory chain complexes I and II, leading to a decrease in ATP production, thereby altering other cellular anabolic metabolisms. At the same time, paclitaxel can promote the expression of spinal nicotinamide adenine dinucleotide phosphate oxidase in neuronal cells, increasing the production of peroxynitrite in nerve cells, indicating that mitochondrial dysfunction and the production of free radicals are closely related to PIPN (20). Paclitaxel can also cause the activation of astrocytes, leading to the release of pro-inflammatory cytokines (such as TNF, interleukin-1β, and interleukin-6), resulting in neuronal sensitization and neurogenic inflammation (21). The results of this study showed that among the 129 patients who were ultimately enrolled and treated with paclitaxel, 65.9% (85/129) had vitamin D deficiency. Patients were divided into vitamin D deficiency group and non-vitamin D deficiency group according to VD levels. The results showed that 59 patients in the VD deficiency group developed PIPN, and 18 patients in the Non-VD deficiency group developed PIPN, with a statistically significant difference. A total of 59.7% (71/129) of patients developed neuropathic pain. When comparing patients divided into NP group and Non-NP group based on the occurrence of neuropathic pain, a significant difference in vitamin D levels was found between the two groups, with the Non-NP group having significantly higher VD levels than the NP group.

Numerous observational studies have indicated a link between vitamin D deficiency and various types of pain, but the causality is not clear. Research has shown that vitamin D can alleviate chronic muscle pain in patients by reducing the levels of inflammatory and pain-related cytokines in the plasma, such as prostaglandin E2 (PGE2), tumor necrosis factor-alpha (TNF-*α*), and leukotriene B4 (LTB4) (22). Additionally, vitamin D is considered a neurotrophic hormone that can improve axonal growth and sensory nerve response in peripheral nerves, promote electrophysiological recovery, and provide neuroprotection by upregulating the expression of the vitamin D receptor (VDR) and downregulating the expression of type I calcium channels (23). By supplementing vitamin D, there can be a significant increase in the expression of nerve growth factor (NGF) in nerve cells, while also reducing blood sugar levels and the expression of inflammatory factors, alleviating peripheral neuropathic pain caused by diabetic peripheral neuropathy (24). Vitamin D can effectively alleviate neuropathic pain (NP), but apart from diabetic neuropathy, there are few clinical studies on the treatment of other types of NP with vitamin D, and there is a lack of high-quality randomized controlled trials, and the specific mechanism of vitamin D in treating NP is still unclear. A retrospective analysis showed that Pretreatment vitamin D insufficiency is associated with a higher risk of NP from paclitaxel, but prospective trials are needed to investigate the correlation between vitamin D and the occurrence of NP, as well as the effectiveness of vitamin D supplementation in preventing NP (25). This experiment used rank correlation analysis to examine the correlation between vitamin D levels and the occurrence of paclitaxel-induced neuropathic pain, and the results showed a negative correlation between the two variables, meaning that the higher the level of vitamin D, the lower the probability of paclitaxel-induced neuropathic pain occurring.

As an important antioxidant in the body, glutathione (GSH) plays a crucial role in maintaining the balance of reactive oxygen species within cells and alleviating oxidative stress damage. The latest research shows that glutathione can act as a ligand for iron (26), binding to excess iron in cells to maintain intracellular iron balance and alleviate oxidative damage to cellular proteins, DNA, and cell membranes. Vitamin D can activate the inherent antioxidant pathways within cells, increase the content of gamma-glutamyl transpeptidase, thereby increasing the level of intracellular glutathione, and provide a certain degree of protection for the integrity of oligodendrocytes and neural conduction pathways, which is vital for the transmission of nerve signals (27). Studies have shown that after paclitaxel chemotherapy, paclitaxel can increase the level of oxidative stress in the dorsal root ganglion (DRG), the dorsal horn of the spinal cord, and the striatum through the silent information regulator 1 (SIRT1)/peroxisome proliferator-activated receptorγ co-activator-1α (PGC-1α) signaling pathway (28), leads to increased neuronal excitability, where extracellular Ca2+ flows inward along a concentration gradient, activating the intrinsic apoptotic pathway, causing neuronal apoptosis, inducing neurotoxicity, and subsequently triggering peripheral neuropathy induced by paclitaxel (PIPN) (29). As a natural antioxidant, glutathione can protect the integrity of oligodendrocytes and nerve conduction pathways and play a vital role in the transmission of nerve information (30, 31). The antioxidant effect of vitamin D is realized in two ways: on one hand, by directly inhibiting the production of nitric oxide synthase to suppress oxidative stress, and on the other hand, by activating the intrinsic antioxidant pathways within the cell, increasing the content of *γ*-glutamyl transpeptidase, thereby raising the level of intracellular glutathione. This plays a certain protective role in the integrity of oligodendrocytes and neural conduction pathways, thus playing a crucial role in the transmission of neural information. In this study, before the start of paclitaxel chemotherapy, the vitamin D-deficient group and the non-deficient group did not receive any external stimuli, so there was no significant difference in glutathione levels between the two groups. However, after paclitaxel chemotherapy, there was a decrease in GSH levels in the serum of patients in both groups to varying degrees, with the preoperative vitamin D-deficient patients showing a more significant decrease compared to those without vitamin D deficiency. At this time, vitamin D levels were positively correlated with GSH levels, which indirectly proves that paclitaxel causes oxidative stress in patients, and those with preoperative vitamin D deficiency have more severe oxidative stress. It is speculated that vitamin D can inhibit oxidative stress in patients, thereby reducing the occurrence of neuropathic pain.

This study intends to assess the occurrence and degree of neuropathic pain in postoperative patients who receive adjuvant chemotherapy with paclitaxel, utilizing the Douleur Neuropathique 4 Questions (DN4) questionnaire. Concurrently, the research will analyze the levels of vitamin D and glutathione in serum to explore the correlation between vitamin D levels and the neuropathic pain induced by paclitaxel chemotherapy, as well as the potential underlying mechanisms. The experimental results also confirmed our hypothesis that there is a certain correlation between vitamin D deficiency and the occurrence of paclitaxel-induced neuropathic pain. In subsequent experiments, In subsequent experiments, we plan to construct mouse models of paclitaxel-induced neuropathic pain with different levels of vitamin D through animal experiments, to further investigate the mechanisms and potential signaling pathways by which vitamin D improves paclitaxel-induced neuropathic pain, thereby enhancing the quality of this experimental study.

In summary, there is a correlation between vitamin D levels and the occurrence of neuropathic pain caused by paclitaxel chemotherapy. Higher levels of vitamin D can significantly reduce the incidence of neuropathic pain, which may be related to the inhibitory effect of vitamin D on oxidative stress levels in the body. This discovery is of significant importance for clinical treatment because it may help doctors better understand the mechanisms of pain during paclitaxel therapy and provide new strategies for the prevention and treatment of such pain. Furthermore, this finding also points the way for future research directions, that is, by modulating vitamin D levels to reduce the neurotoxicity of paclitaxel, thereby improving patients’ quality of life and treatment compliance. Future clinical practice may pay more attention to personalized treatment, adjusting treatment plans by assessing patients’ vitamin D levels to achieve the best therapeutic effects and minimal side effects, which is conducive to improving patients’ treatment experience and clinical prognosis.

## Data Availability

The original contributions presented in the study are included in the article/supplementary material, further inquiries can be directed to the corresponding author.
